# Morbidity in the postoperative follow-up of endoscopic anterior skull base surgery

**DOI:** 10.1016/j.bjorl.2020.02.006

**Published:** 2020-04-11

**Authors:** Gustavo Lara Rezende, Oswaldo Ribeiro Marquez Neto, Selma Aparecida Souza Kückelhaus

**Affiliations:** a Hospital de Base do Distrito Federal, Brasília, DF, Brazil; b Universidade de Brasília, Faculdade de Medicina, Departamento de Morfologia, Brasília, DF, Brazil

**Keywords:** Skull base surgery, Pituitary adenoma, Minimally invasive, Nasal endoscopic surgery

## Abstract

**Introduction:**

Endoscopic access to the sellar region by videoendoscopy shows a low rate of surgical complications, with findings that indicate risk factors for reducing morbidities during and after the postoperative period.

**Objective:**

To evaluate, over a nine-year period, the acquisition of skills by the anterior skull base surgical team, according to the time of elimination of nasal crusts and/or the presence of morbidities in the postoperative follow-up of individuals treated in a tertiary public hospital.

**Methods:**

After confirming the diagnosis of skull base pathologies, the individuals in this study underwent endoscopic surgery according to the rostrocaudal or coronal axis. For the skull base reconstruction, the nasoseptal flap (associated or not with fascia lata with thigh fat) or free graft was used; clinical follow-up of individuals occurred for a minimum period of 12 months. To assess the impact of the surgical approach on patient clinical evolution, qualitative data related to smoking, post-nasal discharge, nasal flow, smell, taste, clinical symptoms of headache, cranial paresthesia, comorbidities and postoperative morbidities were obtained.

**Results:**

The most frequent diagnosis was pituitary macroadenoma (84.14%). The mean absence of crusts in this cohort was 124.45 days (confidence interval 95% = 119.50–129.39). There was a low cerebrospinal fluid fistula rate (3%). Reconstruction with the nasoseptal flap with a fat graft was an independent variable that recorded the highest mean time for the elimination of nasal crusts (=145 days, confidence interval 95% = 127.32–162.68). Allergic rhinitis and smoking were shown to be the most important and independent variables that increased the mean time to eliminate nasal crusts.

**Conclusion:**

The mean time to eliminate nasal crusts did not change over the years during which the procedures were performed, demonstrating the adequate training of the surgical team. Debridement and nasal irrigation with saline solutions should be performed more frequently and effectively in patients with allergic rhinitis, smokers and those who received the nasoseptal flap and fascia lata graft with autologous fat.

## Introduction

Endoscopic access to the sellar region by videoendoscopy is a surgical technique developed in the last two decades that shows a low rate of surgical complications and improves the postoperative quality of life of patients with tumors in the anterior skull base.^1^ The creation of the vascularized nasoseptal flap significantly decreased the hospital length of stay and nasal healing, reducing the excessive use of antibiotics and anti-inflammatory drugs,^2^ in addition to optimizing the expanded surgical access at the base of the skull.

The postoperative period following anterior skull base surgeries require continuous debridement of the nasal cavity to reduce nasal morbidity and intracranial infectious complications^3^; despite the theoretical risk of intracranial contamination by the sinonasal flora, the rate of infections of the central nervous system is low.^4^

There is little data in the medical literature identifying the main risk factors for reducing morbidities during and after the postoperative period. The identification of patients at higher risk for nasal and intracranial morbidity can minimize the discomfort of nasal debridement sessions and perhaps point at better surgical techniques for skull base access and reconstruction. Therefore, the aim of this study was to evaluate, over a nine-year period, the acquisition of skills by the skull base surgical team, regarding the time needed to eliminate nasal crusts and/or the presence of morbidities in the postoperative follow-up of individuals treated at a tertiary public hospital.

## Methods

### Individuals, inclusion and exclusion criteria and ethical aspects

The individuals included in this study were those seen between 2010 and 2018 at Hospital de Base do Distrito Federal, and which, after the confirmation of the diagnosis of skull base pathologies, underwent sinonasal endoscopic surgery for access and reconstruction. The clinical follow-up of the individuals occurred for a minimum period of 1 (one) year after the surgical procedure.

The study included individuals with nasal endoscopic surgery indications due to skull base diseases, who were able to perform the nasal hygiene routine and return for postoperative clinical examination, in addition to being able to answer the questionnaire on clinical symptoms; individuals with residual disease from previous surgeries or chronic nasal inflammation were excluded.

The study was approved by the local ethics committee and was approved at Plataforma Brasil under number CAAE: 13364918000008153.

### Surgical procedures

For a better surgical corridor at the base of the skull, the surgical access routes used were according to the rostrocaudal axis (transcribiform, transplanum, transsellar, with and without middle and transclival turbinectomy) or according to the coronal axis (transnasal to the petrous apex). In all transcribiform, transplanum and transclival accesses, middle turbinectomy and unilateral or bilateral anterior and posterior ethmoidectomy (transcribiform) were performed. In cases of clivus chordoma, a transoral access combined with the endonasal access with fissure and soft palate reconstruction by simple suture were performed.

Most surgeries started by creating the nasoseptal flap in the right nasal cavity using the reverse flap technique,^1^ with its extension being programmed according to the size of the lesions in the preoperative images. In transsellar accesses in individuals with extensive pituitary tumors, the middle turbinectomy on the right was chosen.

To avoid trauma to the mucosa of the upper turbinates, the sphenoid ostia were enlarged and joined medially and laterally. Inferiorly, the sphenoid sinusectomy extended to the sinus floor, with partial resection of the posterior nasal septum and sphenoid rostrum.

For the skull base reconstruction, a nasoseptal flap was used, associated or not with fascia lata and fat, or a free graft from the posterior septal mucosa was employed. The fascia lata was removed together with the adipose tissue of the patient's right thigh and its use was indicated in cases of intraoperative CSF fistulas; in this case the sella turcica was filled with adipose tissue, fitting the edges of the fascia lata posteriorly to its anterior bone opening, and then it was covered with the nasoseptal flap. All patients received bilateral glove finger nasal packing for 24 hours, without the nasal splint.

### Postoperative follow-up

Patient clinical follow-up occurred over a 12-month period after the surgery, weekly in the first month, fortnightly in the second month and monthly from the third month until completing one year. Patient clinical follow-up was interrupted in the absence of crusts in the nasal cavity.

To assess the impact of the surgical approach on the clinical evolution of patients, qualitative data related to post-nasal discharge (0 = absent; 1 = mild; 2 = severe), nasal flow (0 = equal; 1 = better; 2 = worse), smell (0 = preserved; 1 = hyposmia; 2 = anosmia) and taste (1 = present; 2 = absent) were obtained. Also, clinical symptoms of headache, cranial paresthesia, postoperative comorbidities and morbidities were obtained from patients, scoring 1 (one) when present or 2 (two) when absent.

To establish the individuals’ epidemiological profile, data on age, gender and histopathological diagnosis of the lesion were obtained.

### Statistical analysis

**Figure 1:**
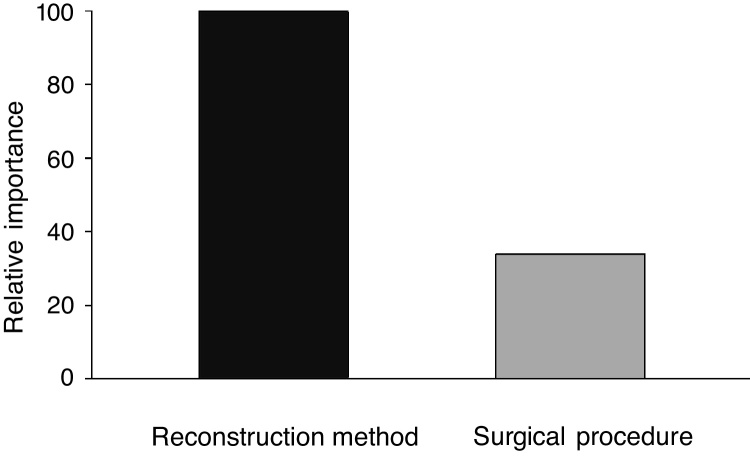
Percentage of the importance of independent variables such as the “reconstruction method” (100%) and type of “surgical procedure” performed (33.85%) in the interaction with the time of elimination of nasal crusts, according to the CART analysis.

**Figure 2:**
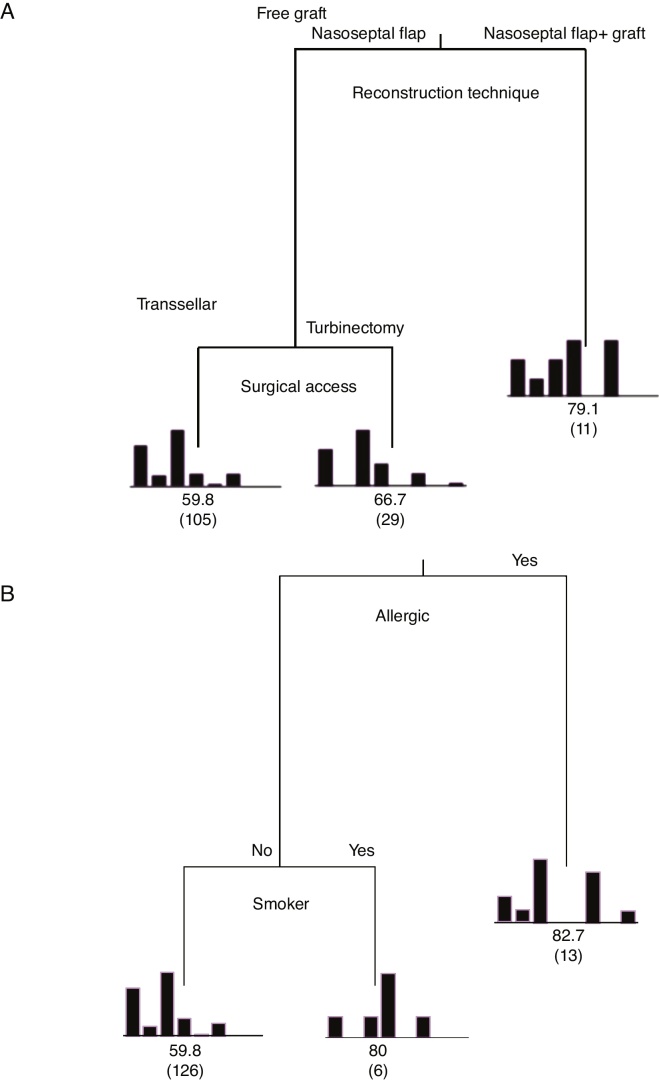
Classification and Regression Tree (CART) for the independent variables regarding the degree of importance on the mean time of elimination of postoperative nasal crusts. A, Patients who used the nasoseptal flap with fascia lata and fat grafts were the ones who had nasal crusts for the longest time (mean of 79.3 days). B, Patients suffering from allergic rhinitis and smokers required an average time to eliminate nasal crusts of 82.7 and 80 days, respectively. Explained variability = 94%.

**Figure 3:**
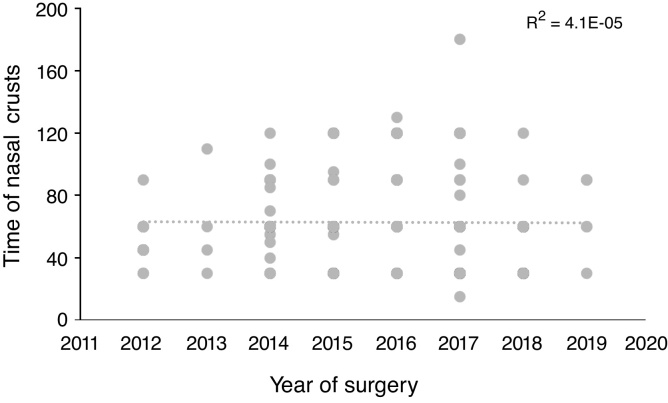
Time of elimination of nasal crusts and year of surgery. The linear regression of the performed learning curve did not show an important statistical association (*R*^2^ = 0.004).

The descriptive and analytical statistical analysis was performed using the SYSTAT 12 software. Descriptive analyses were performed to describe sociodemographic characteristics (gender and age), surgical procedures and reconstruction methods (Table 1), comorbidities (Table 2) and postoperative symptoms (Table 3). Subsequently, classification and regression tree analyses (Classification and Regression Trees – CART) were performed using the TreesPlus software. The univariate Classification and Regression Tree (CART) (only one dependent variable) was used to assess the interaction between the time required for the definitive elimination of nasal crusts (dependent variable) and the independent variables of the surgical techniques and comorbidities (Fig. 1). The analysis also identified the importance of independent variables in each developed model (Fig. 2). The learning curve was performed using the time of nasal crusts and the year in which the surgery was performed using linear regression (Fig. 3). Regarding R2, the closer the value was to 1 (one) represents a positive association.

**Table 1:** Preoperative diagnosis, surgical procedure and mucosal reconstruction method in individuals submitted to skull base surgery.

Diagnosis, *n* (%)	Surgical procedure, *n* (%)	Reconstruction method, *n* (%)
Pituitary macroadenoma, 122 (84%)	Transsellar + middle Turbinectomy 110 (76%)	Nasoseptal flap, 132 (91%)
Functioning microadenoma, 5 (3%)		
Craniopharyngioma, 4 (3%)	Transsellar 29 (21%)	Nasoseptal flap + Fascia Lata/fat, 11 (8%)
Clival chordoma, 3 (2%)	Transplanum 4 (3%)	
Meningioma, clival meningioma, CSF fistula, anaplastic neuroglial cells, Pterygopalatine Schwannoma or petrous apex tumor, 1 (1%)	Transnasal to petrous apex, Transcribiform or Transclival 1 (1%)	Free graft, 2 (1%)

**Table 2:** Comorbidities observed in individuals submitted to endoscopic surgical treatment of skull base tumors.

Comorbidity	Hypertension *n* (%)	Diabetes *n* (%)	Rhinitis *n* (%)	Cushing's disease (%)
No	112 (77.2)	131 (90%)	132 (91%)	136 (94%)
Yes	33 (22.8)	14 (10%)	13 (9%)	9 (6%)

**Table 3:** Nasal symptoms and complications in the postoperative follow-up of individuals submitted to skull base surgery.

*n*	Nasal symptoms and surgical complications, *n* (%)			
1	Nasal discharge	Absent 124 (86%)	Mild 13 (9%)	Severe 8 (6%)
2	Olfaction	Normal 131 (90%)	Hyposmia 11 (8%)	Anosmia 3 (2%)
3	Nasal flow	Normal 119 (82%)	Better 19 (13%)	Worse 7 (5%)
		**Absent**	**Present**	
4	Taste disorder	144 (99%)	1 (1%)	
5	Headache	133 (92%)	12 (8%)	
6	Cranial paresthesia	143 (97%)	2 (1%)	
7	Otitis Media	145 (100%)	–	
8	Nasal voice	144 (99%)	1 (1%)	
9	Velopalatal insufficiency	145 (100)	–	
10	CSF fistula	140 (97%)	5 (4%)	
11	Meningitis	134 (92%)	11 (8%)	
12	Epistaxis	143 (97%)	2 (2%)

## Results

### Individuals’ profile

The study was carried out with 145 patients aged 47.7 ± 17.6 years, of which 79 (54%) were men and 66 (46%) women. The diagnosis, surgical procedures and reconstruction methods are shown in Table 1, while the comorbidities identified in the individuals are shown in Table 2.

### Morbidities at the end of the follow-up

The morbidities identified in the individuals at the end of the study are shown in Table 3. Moreover, two deaths were recorded in the late postoperative period due to intracranial infectious complications.

### Time required for definitive elimination of nasal crusts

The mean time until the absence of crusts in this cohort was 124.45 days (95% CI 119.50–129.39). The mean time until crust absence for the main surgical accesses was 123 days (95% CI 112.92–133.07) in the transsellar procedures and 118 days (95% CI 113.57–124.12) in the transsellar procedures with middle turbinectomy on the right.

Among the reconstruction methods used, the nasoseptal flap showed the shortest mean time until the absence of crusts (=131 days, 95% CI 125.9–136.1) compared to the nasoseptal flap with fascia graft and fat, with 145 days (95% CI 127.3–162.7).

When analyzing the mean time to eliminate postoperative nasal crusts, the independent variable with the greatest importance in the Classification and Regression Tree (CART) model was the “reconstruction technique” of the skull base (100%), followed by the “surgical access” technique, with only 33.8% of relative importance (Fig. 1). Therefore, the Classification and Regression Tree (CART) was created, analyzing the reconstruction technique of the skull base and the type of surgical access. It was observed that patients who underwent the skull base reconstruction with the nasoseptal flap associated with the fascia lata and fat graft, regardless of the type of surgical access, required the longest mean time for the elimination of nasal crusts (=79.1 days) (Fig. 2). This same analysis (CART) between the mean time to eliminate nasal crusts and comorbidities showed that allergic rhinitis was the associated comorbidity that required a longer time for patients to eliminate the postoperative nasal crusts. With an approximate result, patients who were smokers required an average of 80 days to eliminate nasal crusts.

The variables diabetes, hypertension and Cushing's disease showed low relative importance in the model and, therefore, are not represented in the tree (Fig. 2).

The linear regression of the performed learning curve using the time to eliminate nasal crusts and the year in which the surgery was carried out did not show any association (*R*^2^ = 0.004) (Fig. 3).

## Discussion

The prevalence of pituitary macroadenomas in the world's population constitutes four cases per 100,000 individuals.^5^ Although other anterior skull base tumors show similar levels of involvement,^6^ our sample comes from a public health care system that fails to attain an early diagnosis, which contributes to the increase in the prevalence of major lesions, such as pituitary macroadenomas. Asymptomatic and minor lesions often go undiagnosed and lose priority in surgical queue positions.

Skull base reconstruction with vascularized flaps is one of the most reliable ways to reduce CSF fistula rates to below 5%.^7^ We always choose to make the nasoseptal flap regardless of the size of the tumor, which is associated with a low rate of fistulas (3%) in our sample. In most cases, the unpredictability of the occurrence of CSF fistulas and the inevitable trauma to the posterior septal mucosa justify the greater use of this method for skull base reconstruction.

The spectrum of nasal morbidity in the postoperative period of skull base tumors is already well described in the literature.^2^ The post-nasal discharge symptom is more common in patients with chronic sinonasal inflammatory disease who underwent endoscopic surgery. These patients were excluded from this sample, demonstrating the low rate of this complaint in this study.

In a prospective analysis of 775 patients, Pade and Hummel^8^ observed that only 7% to 9% of patients submitted to sinonasal endoscopic surgery had their olfactory capacity reduced. A similar index was reached in our analysis (9.7%), demonstrating the intraoperative care of the surgical technique in preserving the olfactory epithelium of the upper turbinates and posterior nasal septum close to the cribriform plate.

Nasal flow improvement is associated with the automatic correction of septal deviations to access the skull base. Therefore, there was an improvement in the nasal flow perception of 13% in operated patients. Van Zijl et al. (2017)^9^ demonstrated that asymptomatic individuals showed an improvement in the mean NL-NOSE score from 70.0 in the preoperative to 20.0 in the postoperative period in relation to the nasal air flow after the anatomical nasal correction. Thus, septoplasty should always be considered an option for individuals with obstructive deviations regarding quality of life improvement.

The high number of meningitis cases (7%) in this cohort can be justified by the complexity of some tumors with late diagnosis and the difficulty of basic medical assistance to patients with chronic diseases such as diabetes and high blood pressure. In an analysis of 2005 patients, Lai et al.^4^ showed an incidence of meningitis of 1.8% and in those patients who had CSF fistulas, the rate increased to 13%. The independent risk factors for postoperative infection are male gender, previous history of nasal or neurological surgery, peritoneal ventricular shunt and complex intradural procedures,^10^ which did not show any statistical association in this sample.

Although the surgical technique used seems to be a determining factor for the number of days until crust elimination, the reconstruction method showed to be the most predictive of the mean time to eliminate nasal crusts. The use of the nasoseptal flap with a fascia and fat graft at the base of the skull is indicated when there is a diaphragmatic sellar fistula. The predisposition to the occurrence of fistulas increases according to tumor complexity and size, determining a longer surgical time, greater sinonasal manipulation, in addition to the creation of a nasoseptal flap, which exposes a larger area of the nasal septum. Therefore, these factors would increase the local inflammatory healing activity, justifying the greater formation of nasal crusts.

Several reviews^2^, ^11^, ^12^ on the use of autologous fat grafts for the skull base reconstruction have demonstrated high success rates in fistula repairs and mention that the only complication involved were hematomas and seroma at the donor site. Pant et al. (2010)^13^ observed that the mean duration of crusts in the nasal fossa was 126 days, but the use of a nasoseptal flap did not affect the duration of crusts, when compared to cases that did not use the same flap in the skull base reconstruction. Perhaps a limitation of our study would be the failure to include data on the size of the tumor axes, which would facilitate a better understanding of the mechanisms of the sinonasal mucosa epithelialization.

Wound healing is a highly coordinated process that involves the formation of clots, inflammatory reaction, immune response and, finally, tissue remodeling and maturation.^14^ Experimental models of histological analysis of the nasal mucosa demonstrate that exposure to cigarette smoke promotes effects that are opposed to the healing process, such as loss of cellular cilia, fibrosis, goblet cell proliferation and epithelial and subepithelial hypertrophy.^15^ Among several studies involving nasal morbidity in the medical literature,^2^, ^16^, ^17^ few mention allergic rhinitis as a predisposing factor to the greater formation of nasal crusts. In patients with allergic rhinitis and smokers, the use of reverse contralateral nasoseptal flaps should always be considered,^18^ covering the exposed nasal septum after the creation of the nasoseptal flap, decreasing the formation of nasal crusts and minimizing postoperative morbidities. Moreover, postoperative care should be optimized in patients who are smokers and have allergic rhinitis. The advice on irrigation of the nasal cavity with saline solution sprays and antibiotic ointment use should be proposed early in the postoperative period.^19^ Similarly, visits to the medical office for videoendoscopic debridement should start as early as on the 7^th^ postoperative day and followed weekly until the complete elimination of crusts from the nasal cavity.^20^

The average time for crust elimination has not changed over the years after the procedures were performed, demonstrating the adequate training of the surgical team since its beginnings, following international protocols and always updating itself on the technical innovations associated to sinonasal endoscopic surgery for more than a decade. However, we consider it is necessary to improve the access of our population to the early diagnosis of sellar tumors.

## Conclusion

The techniques used for skull base reconstruction showed low rates of CSF fistula. Moreover, nasal debridement and nasal irrigation with saline solution should be more frequent and effective in patients with allergic rhinitis, smokers and those who underwent reconstruction with the nasoseptal flap and fascia lata graft with autologous fat.

## Conflicts of interest

The authors declare no conflicts of interest.
